# Exercise prescription to improve executive functioning in children and adolescents with attention deficit hyperactivity disorder: a network meta-analysis

**DOI:** 10.3389/fpsyt.2026.1716578

**Published:** 2026-02-05

**Authors:** Zixuan Yang, Ke Zhao, Yuanye Hu, Qinhan Zhou

**Affiliations:** 1 Department of Leisure Services Sports, Pai Chai University, Daejeon, Republic of Korea; 2 Department of Physical Education, Zhangjiakou University, Zhangjiakou, Hebei, China

**Keywords:** adolescents, children, executive function, exercise prescription, network meta-analysis

## Abstract

**Objective:**

This study employs a network meta-analysis to investigate the potential effects of exercise type, duration, frequency, intensity, and cycle on executive functions (inhibitory control, working memory, cognitive flexibility) in children and adolescents with ADHD, thereby providing directional insights for future research.

**Methods:**

Five databases were systematically searched up to February 1, 2025, yielding 21 RCTs (n = 1,491) involving participants aged 7–18 years. The risk of bias was assessed using Cochrane tools. Standardised mean differences (SMDs) were used as effect measures, while SUCRA was used for probability ranking and GRADE for evidence quality grading.

**Results:**

Skill-based exercise outperformed isolated aerobic exercise in inhibitory control (SMD = 0.73, 95% CI 0.31–1.41) and cognitive flexibility (SMD = 3.08, 95% CI 0.52–5.63). Combined exercise outperformed controls in working memory (SMD = 0.73, 95% CI 0.35–1.12). SUCRA ranking indicated the highest cumulative probability for skill-based exercise in inhibitory control (95.8) and cognitive flexibility (95.5), while aerobic exercise had the highest probability for working memory (87.1). Sensitivity analyses indicated that estimates for cognitive flexibility were significantly influenced by individual studies, demonstrating limited robustness.

**Conclusion:**

Preliminary evidence suggests that moderate-intensity, skill-based exercise may improve inhibitory control and cognitive flexibility within 6–10 weeks. Aerobic exercise may enhance working memory within 4–5 weeks. However, factors such as ADHD subtypes, age, and dose-response relationships remain unclear. Clinical implementation should be individualised and await high-quality validation.

## 1 Introduction

Attention-Deficit/Hyperactivity Disorder (ADHD) is one of the most common neurodevelopmental problems that affect children’s growth. Its core features include persistent difficulties sustaining attention, deficits in impulse control, and hyperactive-impulsive behaviors. Symptoms may manifest as a single subtype or a combination of multiple features, and are often accompanied by executive function impairments (1). Epidemiological studies indicate a global prevalence of approximately 8.0% among children and adolescents, making it an emerging global public health concern (2). This disorder imposes substantial medical costs on patients and families (3). Additionally, it directly impacts academic achievement and occupational performance, leading to learning difficulties, declining grades, and career setbacks (4), as well as poor sleep quality and non-restorative sleep (5). Executive function (EF) refers to the higher-order cognitive processes required for executing complex tasks (6). Academic consensus identifies three core components: inhibitory control, working memory, and cognitive flexibility (7). This classic framework has been further supported and expanded in subsequent research, with related review studies incorporating “attentional control” (i.e., the ability to actively regulate the direction of attention and maintain focus) into the core system of executive functions, building upon the original three-factor model (8). Research indicates ADHD symptoms stem from impairments in specific EF domains, manifesting as reduced cognitive flexibility, impaired inhibitory control, and compromised working memory (9). In daily life, individuals with impaired executive function often struggle with disorganized time and object management: repeatedly purchasing identical items while shopping; reversing cooking steps; and experiencing persistent procrastination and distractibility during learning or work. These challenges directly impact their quality of life (10).

The primary prescription medication currently used to treat attention-deficit hyperactivity disorder (ADHD) is methylphenidate, which stimulates α and β adrenergic receptors by releasing dopamine and norepinephrine. However, its long-term use is controversial due to potential physical and psychological dependence, increased risk of illicit drug addiction, and even suicide induction. Like commonly abused stimulants such as cocaine, it elevates extracellular dopamine levels in the brain (11). Therefore, improving executive function in Children and adolescents with ADHD and identifying non-pharmacological intervention strategies have become research priorities. Concurrently, exercise therapy has emerged as a new direction in ADHD intervention research due to its ability to modulate patients’ neurological functions (12). By enhancing brain activity—particularly adaptive changes in the prefrontal cortex—it promotes optimization of neural activity patterns, significantly improving executive function in individuals with ADHD (13). In the standardized practice of exercise intervention, various exercise prescription guidelines are grounded in the classic FITT principle—Frequency, Intensity, Time, and Type—providing a standardized framework for the scientific design and implementation of intervention programs (14). Meta-analyses have further demonstrated that exercise intervention significantly improves executive function in children with ADHD, with aerobic exercise showing particularly pronounced effects (15).

Although some studies have employed traditional meta-analysis methods for investigation, conventional meta-analyses typically only combine direct evidence when comparing multiple interventions, making it difficult to systematically integrate the relative effects across all interventions. This approach thus presents limitations in comparing multiple intervention measures. Based on this, network meta-analysis integrates both direct and indirect evidence, enabling not only a more comprehensive assessment of treatment efficacy but also the ranking of all interventions. This provides stronger support for clinical decision-making (16). Furthermore, existing reviews have not thoroughly examined the impact of FITT variables on the executive function of children with ADHD. Therefore, this study employs a network meta-analysis to evaluate different exercise intervention protocols, aiming to provide more diverse exercise recommendations for alleviating executive function deficits in Children and adolescents with ADHD.

## 2 Methods

This study followed the guidelines developed by the Preferred Reporting Items for Systematic Reviews and Meta-Analyses (PRISMA) statement (17). It was registered on the PROSPERO platform with registration number CRD420251016263.(https://www.crd.york.ac.uk/PROSPERO/view/CRD420251016263)

### 2.1 Literature search strategy

A comprehensive search was conducted across five core databases: PubMed, Web of Science, Embase, Scopus, and Cochrane. The search period covered the entire inclusion cycle of each database, spanning from the inception of each database to February 1, 2025. Three types of keywords are used in the search strategy: the first group includes exercise, Strength Training, physical exercise, physical activity, sports, fitness, Functional Training and Exercise Therapy; The second group includes children and adolescents with ADHD, elementary school students, adolescents, young adults, school-aged children, and school-aged people; The third group is executive functioning, working memory, inhibitory control, cognitive flexibility, goal-directed behavior, task switching, short-term memory, persistent retraining, impulse control, response inhibition, interference inhibition, transformative stereotyping, multitasking, RCT, experiment and trial. We use the “AND” of Boolean logic to connect these three groups of words to search. In addition, published references to systematic reviews and meta-analyses were manually checked so that more relevant studies could be found, the specific search strategy is shown in **Table 1**.

**Table 1:** Search strategy.

Number	Databases	Search Strategy
#1	PubMed	((exercise[MeSH Terms] OR "Strength Training"[MeSH Terms] OR "physical exercise"[MeSH Terms] OR "physical activity"[MeSH Terms] OR sports[MeSH Terms] OR fitness[MeSH Terms] OR "Functional Training"[MeSH Terms] OR "Exercise Therapy"[MeSH Terms]) AND ("ADHD"[MeSH Terms] AND children[MeSH Terms] OR "primary school students"[MeSH Terms] OR adolescents[MeSH Terms] OR juvenile[MeSH Terms] OR "school children"[MeSH Terms] OR "school-age population"[MeSH Terms]) AND ("Executive Function"[MeSH Terms] OR "Working Memory"[MeSH Terms] OR "Inhibitory Control"[MeSH Terms] OR "Cognitive Flexibility"[MeSH Terms] OR "Goal-Directed Behavior"[MeSH Terms] OR "Task Switching"[MeSH Terms] OR "Short-Term Memory"[MeSH Terms] OR "Maintenance Rehearsal"[MeSH Terms] OR "Impulse Control"[MeSH Terms] OR "Response Inhibition"[MeSH Terms] OR "Interference Inhibition"[MeSH Terms] OR "Set Shifting"[MeSH Terms] OR "Multitasking"[MeSH Terms] OR "RCT"[MeSH Terms] OR "experiment"[MeSH Terms] OR "trial"[MeSH Terms]))
#2	Web of science	TS=(exercise OR "Strength Training" OR "physical exercise" OR "physical activity" OR sports OR fitness OR "Functional Training" OR "Exercise Therapy") AND TS=("ADHD children" OR "primary school students" OR adolescents OR juvenile OR "school children" OR "school-age population") AND TS=("executive functions" OR "working memory" OR "inhibitory control" OR "cognitive flexibility" OR "goal - directed behavior" OR "task - switching" OR "short - term memory" OR "maintenance rehearsal" OR "impulse control" OR "response inhibition" OR "interference inhibition" OR "set - shifting" OR "multitasking" OR RCT OR experiment OR trial)
#3	Embase	1. (exercise OR "Strength Training" OR "physical exercise" OR "physical activity" OR sports OR fitness OR "Functional Training" OR "Exercise Therapy") [emtree]/exp 2. ("ADHD children" OR "primary school students" OR adolescents OR juvenile OR "school children" OR "school-age population") [emtree]/exp 3. ("executive functions" OR "working memory" OR "inhibitory control" OR "cognitive flexibility" OR "goal - directed behavior" OR "task - switching" OR "short - term memory" OR "maintenance rehearsal" OR "impulse control" OR "response inhibition" OR "interference inhibition" OR "set - shifting" OR "multitasking" OR RCT OR experiment OR trial) [emtree]/exp 4. 1 AND 2 AND 3
#4	Scopus	1. TITLE-ABS-KEY(exercise OR "Strength Training" OR "physical exercise" OR "physical activity" OR sports OR fitness OR "Functional Training" OR "Exercise Therapy") 2. TITLE-ABS-KEY("ADHD children" OR "primary school students" OR adolescents OR juvenile OR "school children" OR "school-age population") 3. TITLE-ABS-KEY("executive functions" OR "working memory" OR "inhibitory control" OR "cognitive flexibility" OR "goal - directed behavior" OR "task - switching" OR "short - term memory" OR "maintenance rehearsal" OR "impulse control" OR "response inhibition" OR "interference inhibition" OR "set - shifting" OR "multitasking" OR RCT OR experiment OR trial) 4. 1 AND 2 AND 3
#5	Cochrane	1. (exercise OR "Strength Training" OR "physical exercise" OR "physical activity" OR sports OR fitness OR "Functional Training" OR "Exercise Therapy") [MeSH Terms] 2. ("ADHD children" OR "primary school students" OR adolescents OR juvenile OR "school children" OR "school-age population") [MeSH Terms] 3. ("executive functions" OR "working memory" OR "inhibitory control" OR "cognitive flexibility" OR "goal - directed behavior" OR "task - switching" OR "short - term memory" OR "maintenance rehearsal" OR "impulse control" OR "response inhibition" OR "interference inhibition" OR "set - shifting" OR "multitasking" OR RCT OR experiment OR trial) [MeSH Terms] 4. 1 AND 2 AND 3

### 2.2 Inclusion exclusion criteria

The selection criteria for this study were based on the PICOS framework. To be included, these conditions must be met: (1) the participants were young people aged 7 to 18 years old with a diagnosis of ADHD; (2) In this study, the inclusion criteria encompassed research involving any form of exercise intervention, with no mandatory requirements for the type, duration, or frequency of exercise, but the intervention period must be no less than 4 weeks, and the control group was a passive control group that did not receive any form of physical activity intervention, maintaining only their daily routine;(3) the research method must be a randomized controlled trial (RCT);(4) the results measured should be important executive functions, especially inhibitory control, working memory, and cognitive flexibility.

Studies will be excluded if they: (1) are not experimental, such as cross-sectional, observational, or descriptive studies;(2) are not original studies, such as systematic reviews, meeting summaries, newsletter reviews, or reports with insufficient intervention details;(3) lack key data and cannot be obtained from other sources.

### 2.3 Data extraction

Endnote 20.0 software was used in this study to remove duplicate literature. Subsequently, the remaining study titles and abstracts were screened by two researchers in strict adherence to the inclusion and exclusion criteria, and the inclusion of studies was assessed based on the pre-established inclusion criteria, and eligible studies were entered into the full-text search stage with further eligibility assessment, followed by the analysis stage. The following information extraction was then completed: first author of the literature, year of publication, sample characteristics, exercise prescription parameters (type, period, frequency, intensity), and core outcome indicators (inhibitory control, working memory, cognitive flexibility). In case of inconsistency in coding results, the results were independently cross-checked by the above two researchers, and in case of disagreement, the decision was made after the judgment of a third party.

### 2.4 Assessment of risk of bias in included studies

In this study, we determined the risk of bias by reviewing the ROSPERO registration information of all included articles and independently used the Cochrane Risk of Bias Assessment Tool (18) to assess them. The evaluation process focuses on seven key aspects: whether randomization is sufficient, whether participants and investigators are blinded, whether result evaluators are blinded, whether allocation concealment procedures are in place, whether the result data is complete and accurate, whether there is a possibility of selectively reporting results, and other possible sources of bias. Each study was subsequently classified as low risk, high risk, or uncertain quality risk. If there are differences during the evaluation process, the evaluator will resolve them through discussion. If agreement cannot be reached, the lead researcher will make the final decision after considering the majority opinion and his own judgment.

### 2.5 Statistical methods

The statistical analysis of this survey was completed using Stata 17.0. Because the results of all major studies are continuous variables and use different evaluation tools and units, we use the Standardized Mean Difference (SMD) and its 95% confidence interval as the effect size indicator for data synthesis. A positive SMD indicates an improvement in executive function, while a negative SMD signifies the opposite effect. After conducting global and local inconsistency tests, the system evaluates the results of direct and indirect comparisons of different interventions. If there was no significant heterogeneity between direct and indirect evidence (P > 0.05), we used a consistency model to synthesize the effect sizes (19). To further determine the treatment rankings of different interventions, we used the SUCRA (Area Under the Cumulative Ranking Curve) technique. SUCRA is a web-based meta-analysis tool that compares the relative effects of all possible interventions. SUCRA values range from 0 to 100, with higher scores indicating better relative treatment outcomes in ADHD management. Finally, we used a corrected comparison funnel plot to assess publication bias.

### 2.6 Evidence certainty assessment

In this survey, we mainly used the GRADE framework to evaluate the credibility of each study result (20). Specifically, the assessment results showed that there were 3 pieces of high-level evidence, which indicated that the study design and implementation of these outcome indicators were of high quality, and the results were more reliable and could provide a more solid basis for clinical decision-making. There were 11 pieces of intermediate-level evidence, which, although it may have some limitations in certain aspects, still has a certain reference value. There were 6 low-level evidence, and these studies may have more methodological flaws or insufficient data, resulting in less credible results. In addition, there is 1 very low level of evidence, which suggests that there are serious problems with the quality of the studies on this outcome indicator and that the results are more uncertain and need to be treated with caution. In grading the quality of the evidence, we found that limitations were the main factor that led to the downgrading of the evidence. Specifically, 18 studies were downgraded because of methodological limitations. These limitations may include flaws in study design, inadequate sample size, poor data collection methods, lack of blinding, or randomization. All of these factors may affect the accuracy and reliability of the study results, leading to a reduction in the quality of the evidence. (See **Table 2**).

**Table 2:** Evaluation of the quality of evidence in the included literature.

Author & year	Limitations	Inconsistency	Indirectness	Imprecision	Publication bias	Level of evidence
Verret et al,2012 (21)	-1	0	0	0	0	Moderate
Ziereis et al,2015 (22)	-1	0	0	0	0	Moderate
Ji et al,2023 (23)	-1	0	0	0	0	Moderate
Kadri et al,2019 (24)	-1	0	0	0	0	Moderate
Memarmoghaddam et al,2016 (25)	-1	0	0	-1	0	Low
Hoza et al,2015 (26)	-1	0	0	0	0	Moderate
Bustamante et al,2016 (27)	-1	-1	-1	0	0	Very Low
Choi et al,2015 (28)	-1	0	-1	0	0	Low
Chou et al,2017 (29)	-1	0	0	0	0	Moderate
Benzing et al,2019 (30)	-1	-1	0	0	0	Low
Liang et al,2022 (31)	0	0	0	0	0	High
Jensen et al,2004 (32)	-1	0	0	0	0	Moderate
Nejati et al,2021 (33)	-1	-1	0	0	0	Low
Pan et al,2016 (34)	-1	-1	0	0	0	Low
Silva et al,2020 (35)	-1	0	0	0	0	Moderate
Berg et al,2019 (36)	-1	0	0	0	0	Moderate
Hattabi et al,2019 (37)	-1	0	0	0	0	Moderate
Rezaei et al,2018 (38)	0	0	0	0	0	High
Chang et al,2022 (39)	0	-0	0	0	0	High
Li et al,2025 (40)	-1	0	0	0	0	Moderate
Ludyga,2022 (41)	-1	-1	0	0	0	Low

## 3 Results

### 3.1 Results of literature search

**Figure 1:**
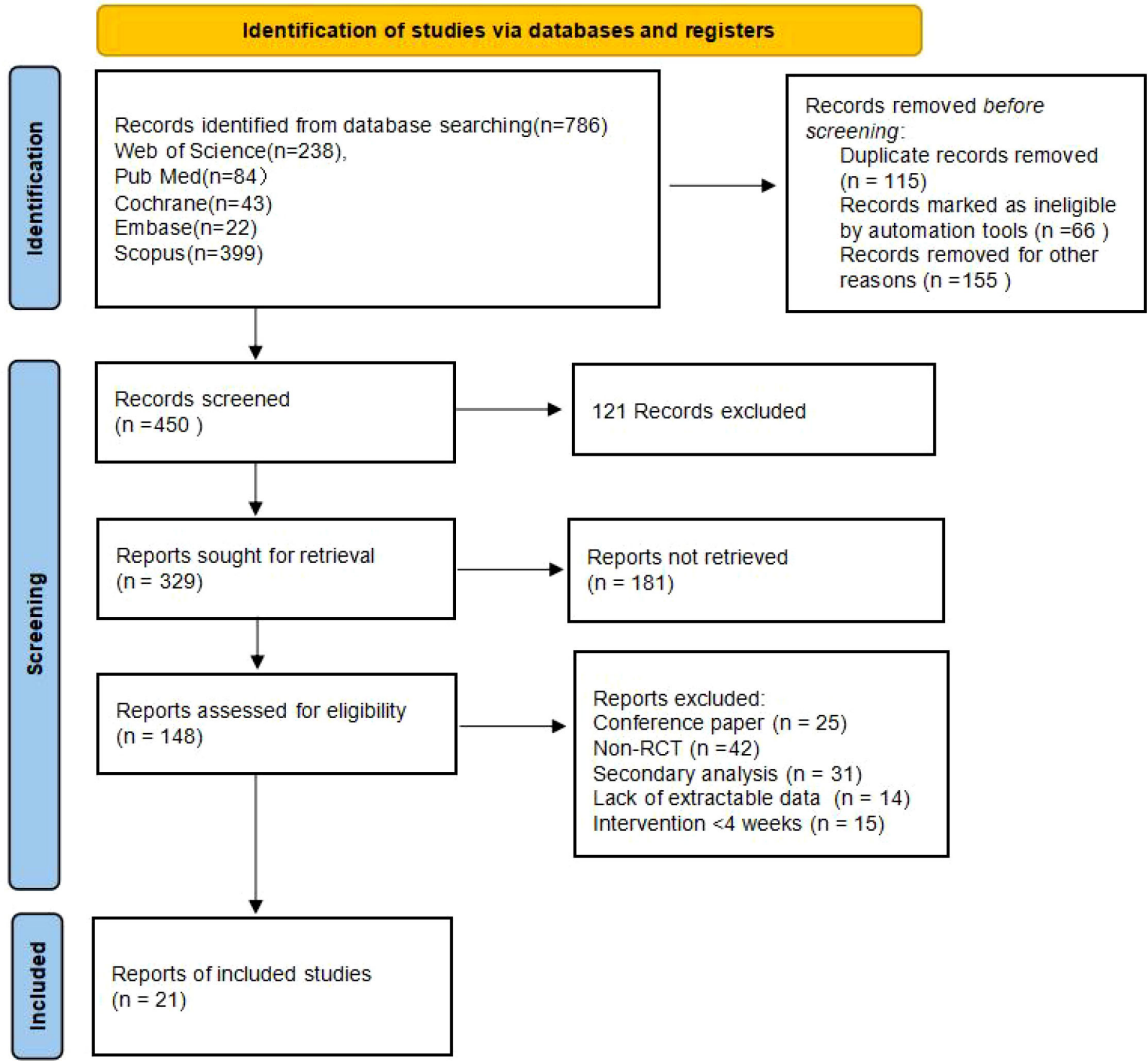
PRISMA flow diagram of the study process.

During the literature search, 1572 records were initially obtained. With the help of EndNote 20.0 literature management tool, 629 duplicates were excluded from the process of de-duplication. Based on reading the title and abstract information, 486 documents with insufficient subject fit were screened out. After supplementing 25 related documents through citation retrospection, 461 documents that did not meet the inclusion criteria were excluded after full-text accessibility verification. Finally, an analyzed sample set consisting of 21 literature was formed, and the complete screening process is detailed in **Figure 1**.

### 3.2 Basic characteristics of included studies

This study included a total of 21 articles, encompassing 1,491 participants aged 7–18 years, all of whom were minors diagnosed with attention-deficit hyperactivity disorder. Based on the core characteristics of exercise and considering the physical and mental development laws of children and adolescents, the intervention programs were systematically categorized into three types: Combined Exercise (CE), which refers to comprehensive training that integrates two or more exercise modes, such as exercise combined with cognition or Exergame; Aerobic Exercise (AE), which involves endurance sports activities performed under sufficient oxygen supply, such as jogging or those explicitly identified as aerobic exercise in the research. Skill-based exercise (SE), which aims to enhance specific movement skills, coordination, precision, or specialized abilities, such as judo, yoga, and coordination exercises (42, 43). The exercise intensity is defined as follows: moderate intensity corresponds to 55%–70% of HRmax; moderate-to-high intensity starts at 55%–75% of HRmax and gradually increases to ≥75%–85% of HRmax during the intervention period (44). The detailed characteristics of the included literature are presented in **Table 3**.

**Table 3:** Basic characteristics of included studies.

Author & year	Country	Sample size		Mean age (years)		Instrument	Dose
		E	C	E	C		
Verret et al,2012 (21)	Canada	10	11	9.10 ± 1.1 0		AE	45min,3times/week,10weeks,moderate intensity
Ziereis et al,2015 (22)	Germany	13/14	16	9.20 ± 1.30	9.50 ± 1.40	AE/CE	60min,1time/week,12weeks
Ji et al,2023 (23)	South Korea	16	14	10.50 ± 1.20		CE	20min,2times/week,4weeks, moderate intensity
Kadri et al,2019 (24)	Tunisia	20	20	14.0 ± 3.50	14.20 ± 3.00	SE	50min,2times/week,5weeks,moderate intensity
Memarmoghaddam et al,2016 (25)	Iran	19	17	8.31 ± 1.29	8.29 ± 1.31	AE	90min,3times/week,8weeks,moderate intensity
Hoza et al,2015 (26)	United States	94	108	6.83 ± 0.96		AE	31min,5times/week,8weeks,medium to high intensity
Bustamante et al,2016 (27)	United States	19	16	9.40 ± 2.20	8.70 ± 2.00	CE	90min,5times/week,10weeks,moderate intensity
Choi et al,2015 (28)	South Korea	13	17	15.0 ± 1.70	16.0 ± 1.20	AE	90min,3times/week,6weeks,moderate intensity
Chou et al,2017 (29)	China	25	25	10.7± 1.00	10.30 ± 1.07	SE	40min,2times/week,8weeks,moderate intensity
Benzing et al,2019 (30)	Switzerland	28	23	10.4 ± 1.30	10.3 ± 1.44	CE	30min,3times/week,8weeks
Liang et al,2022 (31)	China	40	40	8.37 ± 1.42	8.29 ± 1.27	CE	60min,3times/week,12weeks,medium to high intensity
Jensen et al,2004 (32)	Australia	11	8	10.6 ± 1.78	9.35 ± 1.70	CE	60min,1time/week,20weeks,moderate intensity
Nejati et al,2021 (33)	Iran	15	15	9.43 ± 1.43		CE	40-50min,3times/week,4-5weeks,moderate intensity
Pan et al,2016 (34)	China	16	16	8.93 ± 1.49	8.87 ± 1.56	SE	70min,2times/week,12weeks,moderate intensity
Silva et al,2020 (35)	Brazil	18	15	12.0 ± 2.00	12.0 ± 1.00	SE	45min,2times/week,8weeks,moderate intensity
Berg et al,2019 (36)	Netherlands	263	249	10.50± 1.30		CE	10min,5times/week,9weeks,medium to high intensity
Hattabi et al,2019 (37)	Tunisia	20	20	9.95 ± 1.31	9.75 ± 1.33	SE	90min,3times/week,12weeks,moderate intensity
Rezaei et al,2018 (38)	Iran	7	7	9.10 ± 1.30		SE	45min,3times/week,8weeks,moderate intensity
Chang et al,2022 (39)	China	16	16	8.31 ± 1.30	8.38 ± 1.20	SE	60min,3times/week,12weeks,moderate intensity
Li et al,2025 (40)	China	60	60	8.40 ± 1.30		SE	30min,3times/week,12weeks,medium to high intensity
Ludyga,2022 (41)	Switzerland	23	18	10.0 ± 1.2	10.8 ± 1.2	SE	60min,3times/week,12weeks,moderate intensity

E=Experimental Group; C, Control Group; CE, Combined Exercise; AE, Aerobic Exercise; SE, Skill-based Exercise.

### 3.3 Quality assessment of included literature

**Figure 2:**
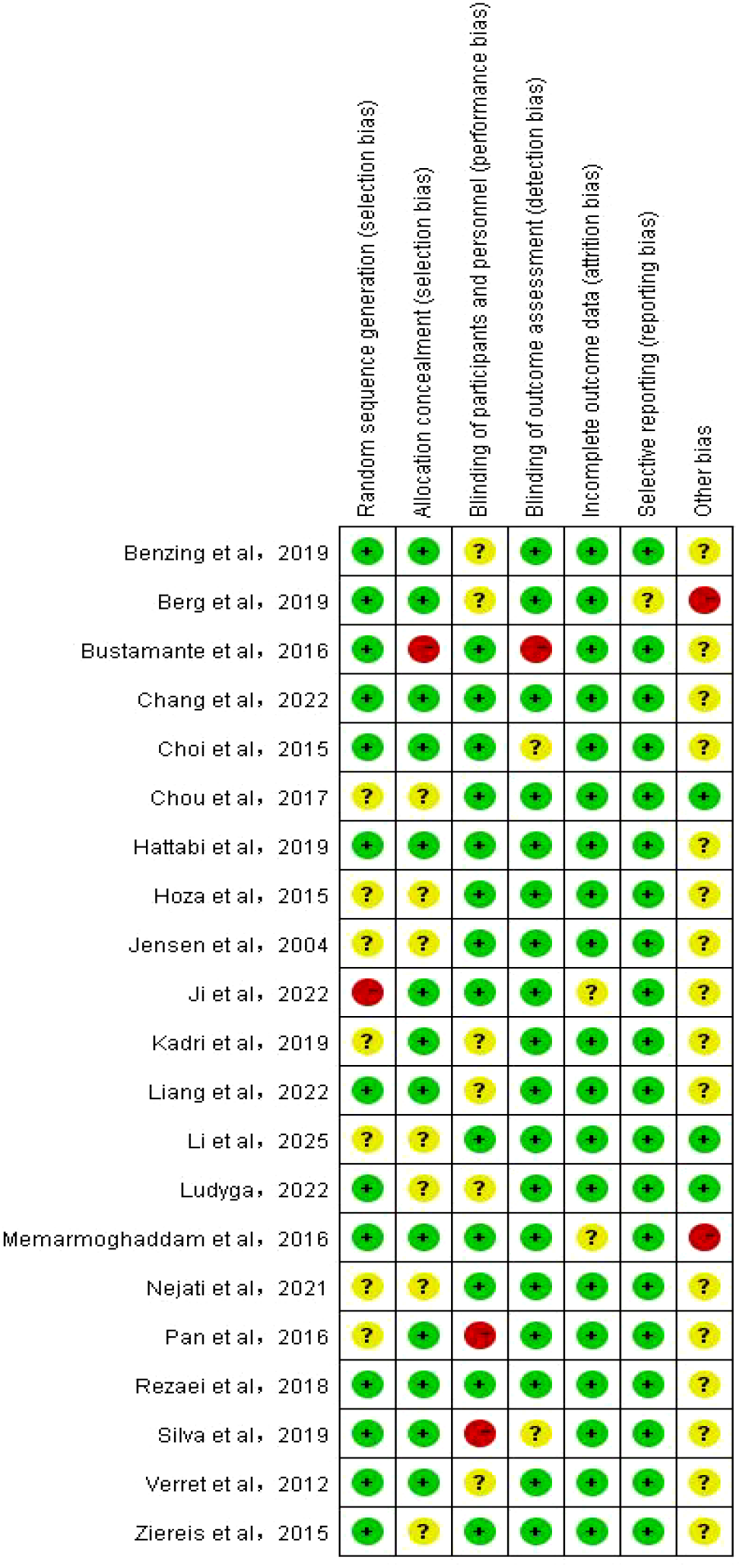
Risk of bias summary.

**Figure 3:**
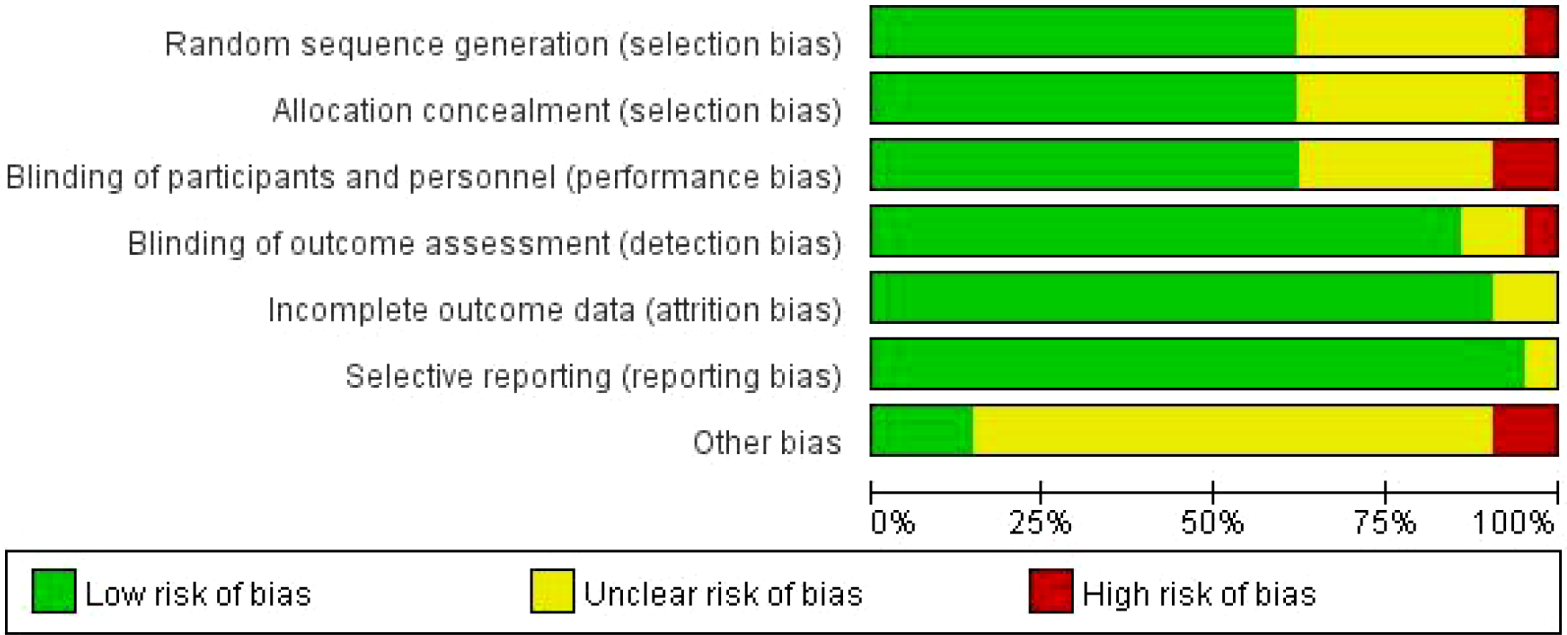
Risk of bias graph.

RevMan 5.4 software was used exclusively for generating risk of bias plots, conducting a comprehensive and rigorous assessment of the quality of included studies. Stata was employed for statistical analysis (45). The specific risk of bias assessment results for each study are detailed in **Figure 2**, while **Figure 3** visually presents the overall distribution characteristics of the risk of bias across all included studies. Green indicates low risk of bias, yellow represents uncertain risk of bias, and red denotes high risk of bias. Studies that use randomization are considered low-risk in the assessment of selection bias; conversely, studies that do not use randomization or do not explain how randomization is considered high-risk.However, among numerous studies, only a handful have successfully implemented blinding involving both participants and therapists. This is primarily due to the numerous challenges associated with executing double-blind procedures in non-pharmacological research, which increases the risk of bias. Detailed results of the study’s bias risk assessment are presented in **Table 4**.

**Table 4:** Risk of bias assessment (n=21).

Author & Year	Random sequence generation (selection bias)	Allocation concealment (selection bias)	Blinding of patients and personnel (performancebias)	Blinding of outcome assessment (detection bias)	Incomplete outcome data (attrition bias)	Selective outcome reporting (reporting bias)	Any other bias
Verret et al,2012 (21)	Low	Low	Unclear	Low	Low	Low	Unclear
Ziereis et al,2015 (22)	Low	Unclear	Low	Low	Low	Low	Unclear
Ji et al,2023 (23)	High	Low	Low	Low	Unclear	Low	Unclear
Kadri et al,2019 (24)	Unclear	Low	Unclear	Low	Low	Low	Unclear
Memarmoghaddam et al,2016 (25)	Low	Low	Low	Low	Unclear	Low	High
Hoza et al,2015 (26)	Unclear	Unclear	Low	Low	Low	Low	Unclear
Bustamante et al,2016 (27)	Low	High	Low	High	Low	Low	Unclear
Choi et al,2015 (28)	Low	Low	Low	Unclear	Low	Low	Unclear
Chou et al,2017 (29)	Unclear	Unclear	Low	Low	Low	Low	Low
Benzing et al,2019 (30)	Low	Low	Unclear	Low	Low	Low	Unclear
Liang et al,2022 (31)	Low	Low	Unclear	Low	Low	Low	Unclear
Jensen et al,2004 (32)	Unclear	Unclear	Low	Low	Low	Low	Unclear
Nejati et al,2021 (33)	Unclear	Unclear	Low	Low	Low	Low	Unclear
Pan et al,2016 (34)	Unclear	Low	High	Low	Low	Low	Unclear
Silva et al,2020 (35)	Low	Low	High	Unclear	Low	Low	Unclear
Berg et al,2019 (36)	Low	Low	Unclear	Low	Low	Unclear	High
Hattabi et al,2019 (37)	Low	Low	Low	Low	Low	Low	Unclear
Rezaei et al,2018 (38)	Low	Low	Low	Low	Low	Low	Unclear
Chang et al,2022 (39)	Low	Low	Low	Low	Low	Low	Unclear
Li et al,2025 (40)	Unclear	Unclear	Low	Low	Low	Low	Low
Ludyga,2022 (41)	Low	Unclear	Unclear	Low	Low	Low	Low

### 3.4 Results of network meta-analysis

**Figure 4:**
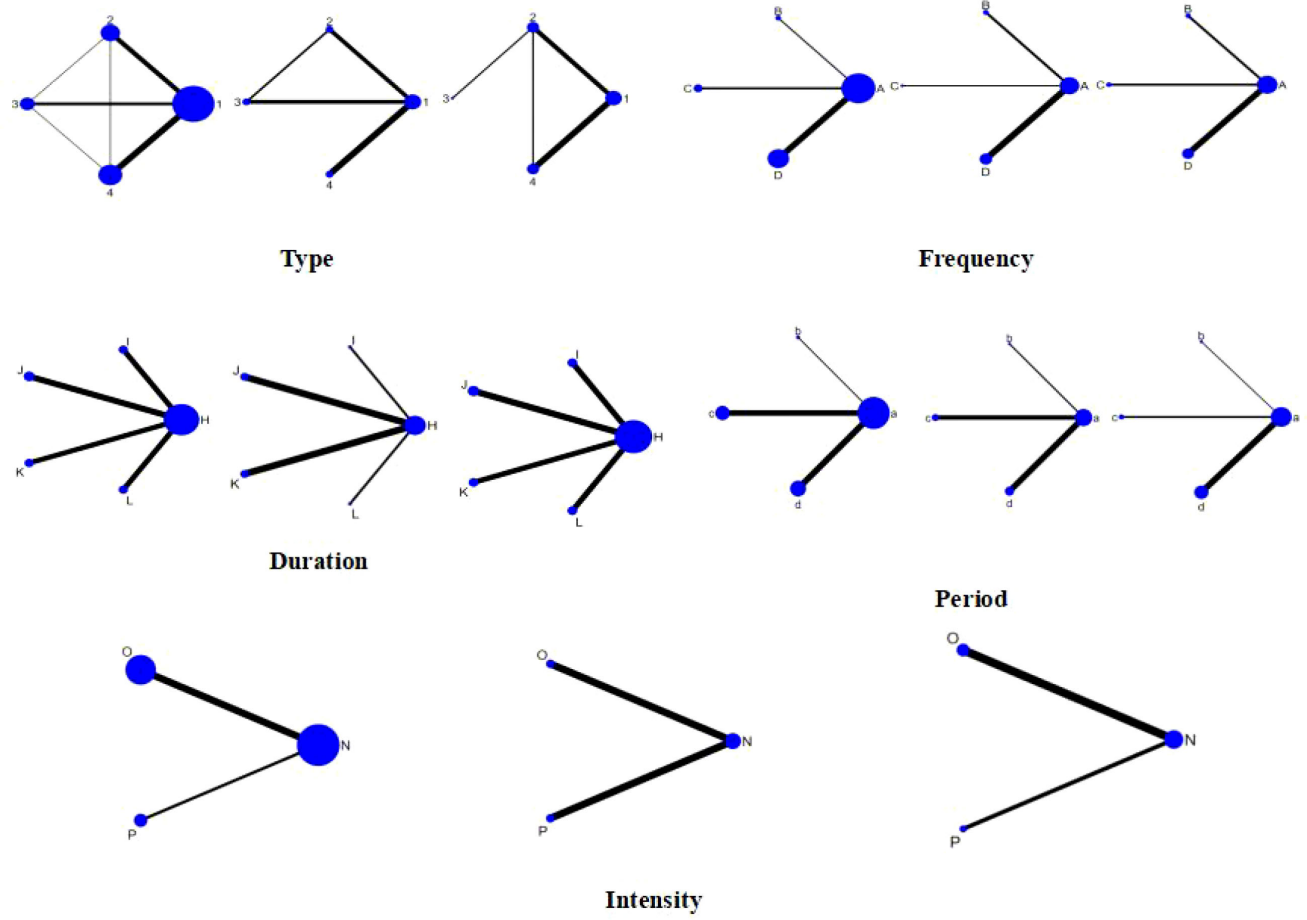
Network evidence diagram. 1, con; 2, Combined Exercise; 3, Aerobic Exercise; 4, Skill-based Exercise; A, con; B, 1time; C, 2times; D, 3-5times; H, con; I,10-31min; J, 40-50min; K,60min; L,≥70min; a, con; b,4-5weeks; c, 6-10weeks; d, ≥12weeks; N, con; O, Moderate intensity; P, Medium to high intensity.

As illustrated in the network structure of **Figure 4**, lines connecting nodes indicate direct comparative relationships between corresponding interventions, while the absence of lines signifies no direct comparative RCTs between studies. Indirect comparative relationships may then be employed for network meta-analysis comparisons, with wider lines denoting a higher frequency of comparative studies between the two interventions. Each vertex represents a distinct intervention, with circle area proportional to the number of participants in included studies. Solid lines denote at least one head-to-head trial, while dashed segments (e.g., Skill-based vs Combined in inhibitory control and cognitive flexibility dimensions) indicate current pairwise comparisons rely entirely on indirect pathways, carrying high estimation uncertainty. No direct links exist between skill-based exercise, single aerobic exercise, and combined exercise across both inhibitory control and cognitive flexibility outcomes. Indirect evidence contributes 100% to the findings, with the narrowest line segments indicating network sparsity and dominance of the transitivity hypothesis.

#### 3.4.1 Inconsistency test

In network meta-analysis, model consistency reflects the alignment between direct and indirect evidence, with higher statistical values indicating greater reliability (46). Global inconsistency testing revealed good consistency in the pooled effects of exercise interventions on core executive function outcomes (inhibitory control, working memory, cognitive flexibility) (P = 0.48, 0.49, 0.85). Further testing of closed loops revealed that the lower confidence limits of inconsistency factors for all intervention methods included zero, indicating strong loop consistency, Local inconsistencies were assessed using the node splitting method. All results showed P > 0.05, indicating no significant inconsistencies were observed in the study area. Consequently, the consistency model was selected for effect size integration. For other interventions (e.g., duration per session, weekly frequency, and cycle), no closed-loop evidence structures formed, thus precluding the need for local consistency verification (47). The network evidence diagram visually presents direct comparative evidence and indirect linkage pathways between different interventions by illustrating connections between nodes.

#### 3.4.2 Results of two-by-two comparison between elements of exercise prescription

**Figure 5:**
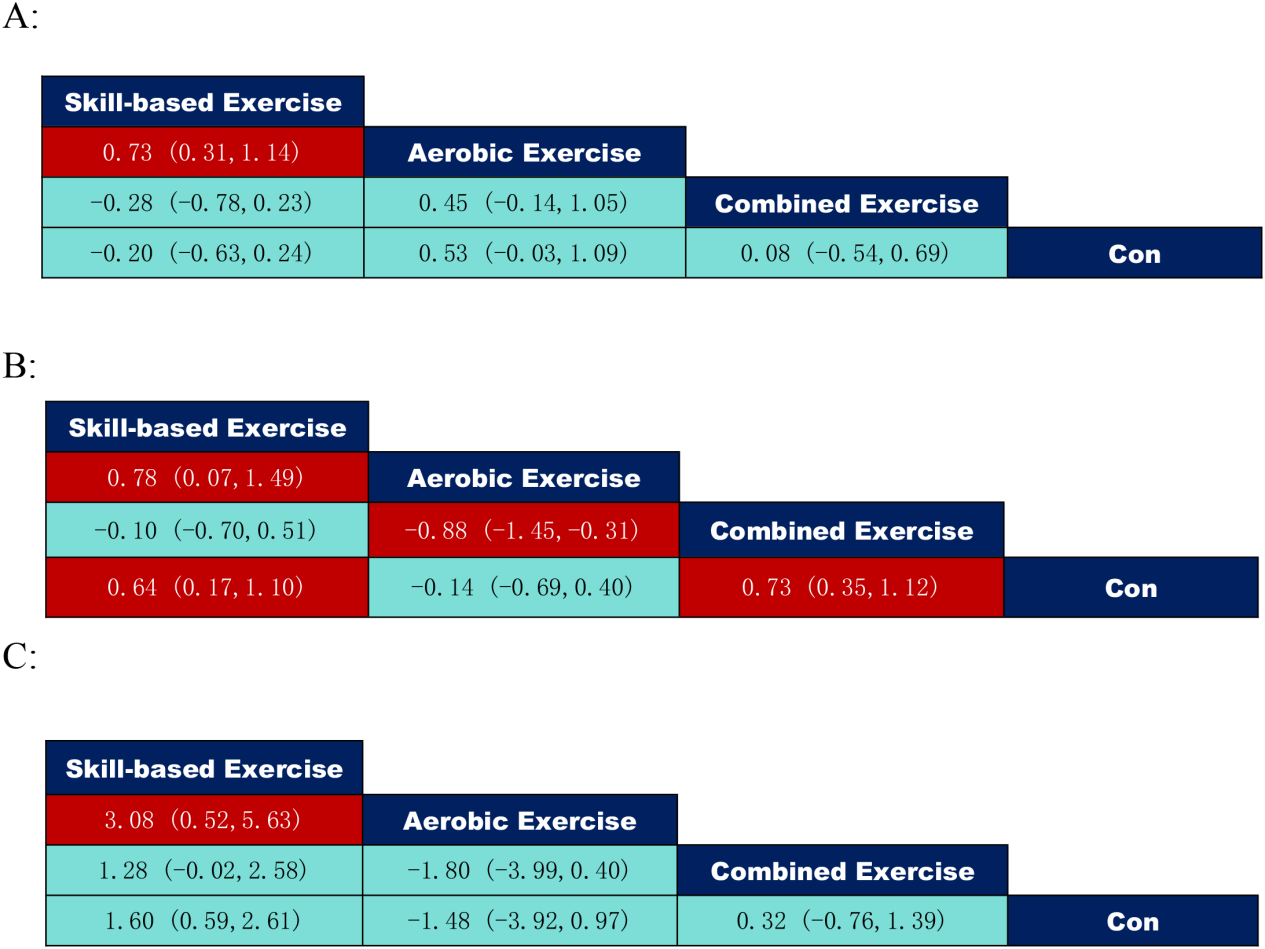
League table of pairwise comparisons of intervention effects among exercise type elements. **(A)** Inhibitory control; **(B)** Working memory; **(C)** Cognitive flexibility.

According to the data of the exercise intervention modalities in **Figure 5**, where inhibitory control: skill-based exercise (SMD = 0.73, 95% CI: (0.31,1.41)) was significantly better than aerobic exercise; working memory: combined exercise (SMD = 0.73, 95% CI: (0.35,1.12)) was significantly better than the control group, aerobic exercise (SMD=-0.88, 95% CI: (-1.45,-0.31)) was significantly weaker than combined exercise, skill-based exercise (SMD = 0.64, 95% CI: (0.17,1.10)) was significantly better than control, and skill-based exercise (SMD = 0.78, 95% CI: (0.07,1.49)) was also significantly better than aerobic exercise; cognitive flexibility: skill-based exercise (SMD = 3.08, 95% CI: (0.52, 5.63)) may be superior to isolated aerobic exercise.

**Figure 6:**
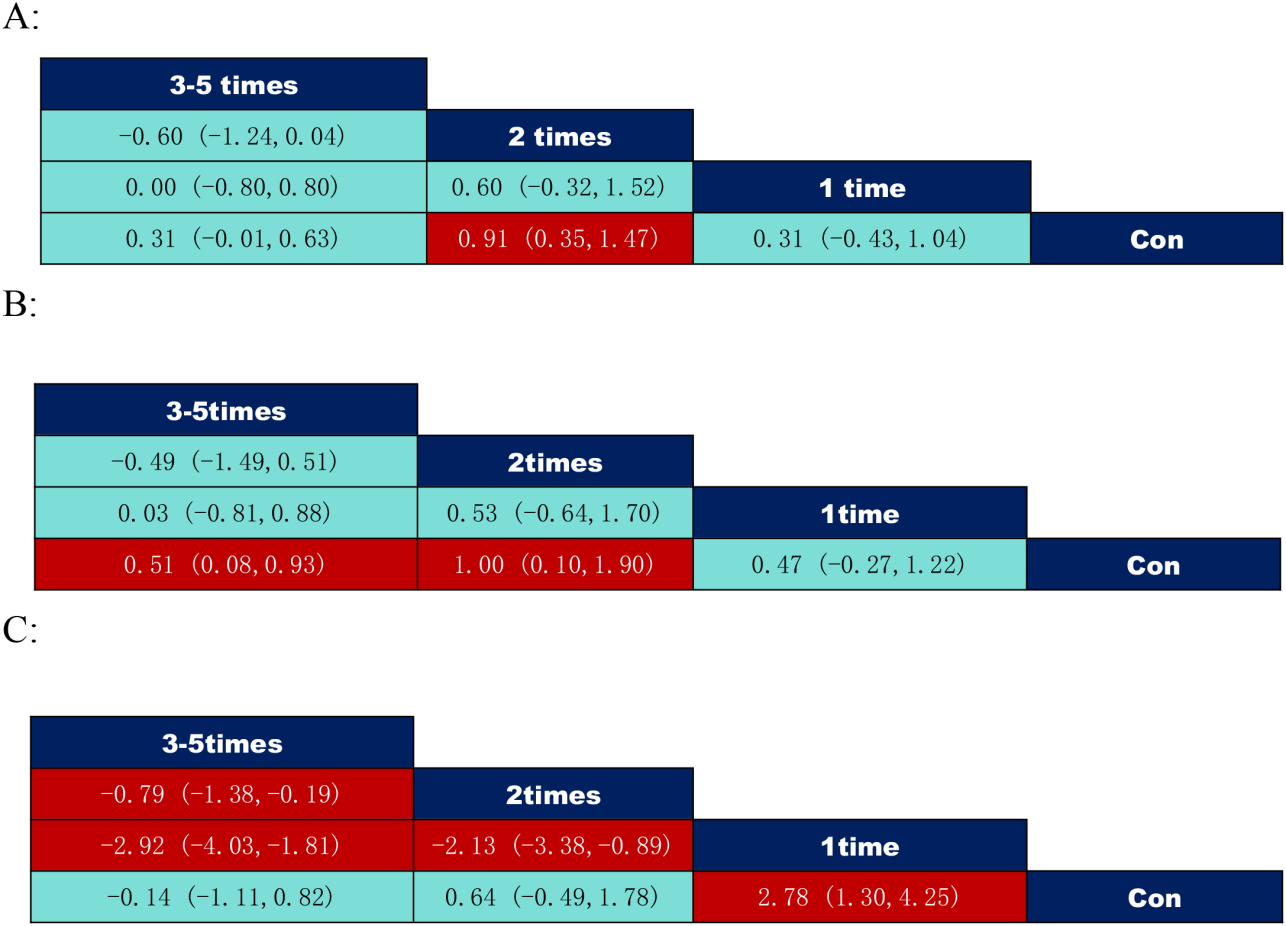
League table of pairwise comparisons of intervention effects among exercise frequency elements. **(A)** Inhibitory control; **(B)** Working memory; **(C)** Cognitive flexibility.

According to the data on the number of exercise interventions in **Figure 6**, among them inhibitory control: 2 times per week (SMD = 0.91, 95% CI: (0.35,1.47)) was significantly better than the control group; working memory: 2 times per week (SMD = 1.00, 95% CI: (0.10,1.90)) was significantly better than the control group, 3–5 times per week (SMD = 0.51, 95% CI: (0.08,0.93)) significantly better than controls; cognitive flexibility: 1 time per week (SMD = 2.78, 95% CI: (1.30,4.25)) significantly better than controls, 2 times per week (SMD=-2.13, 95% CI: (-3.38,-0.89)) significantly weaker than 1 time per week intervention. 3–5 times per week (SMD=-0.79, 95% CI: (-1.38,-0.19)) was significantly weaker than 2 times per week and 1 time per week intervention (SMD=-2.92, 95% CI: (-4.03,-1.81)).

**Figure 7:**
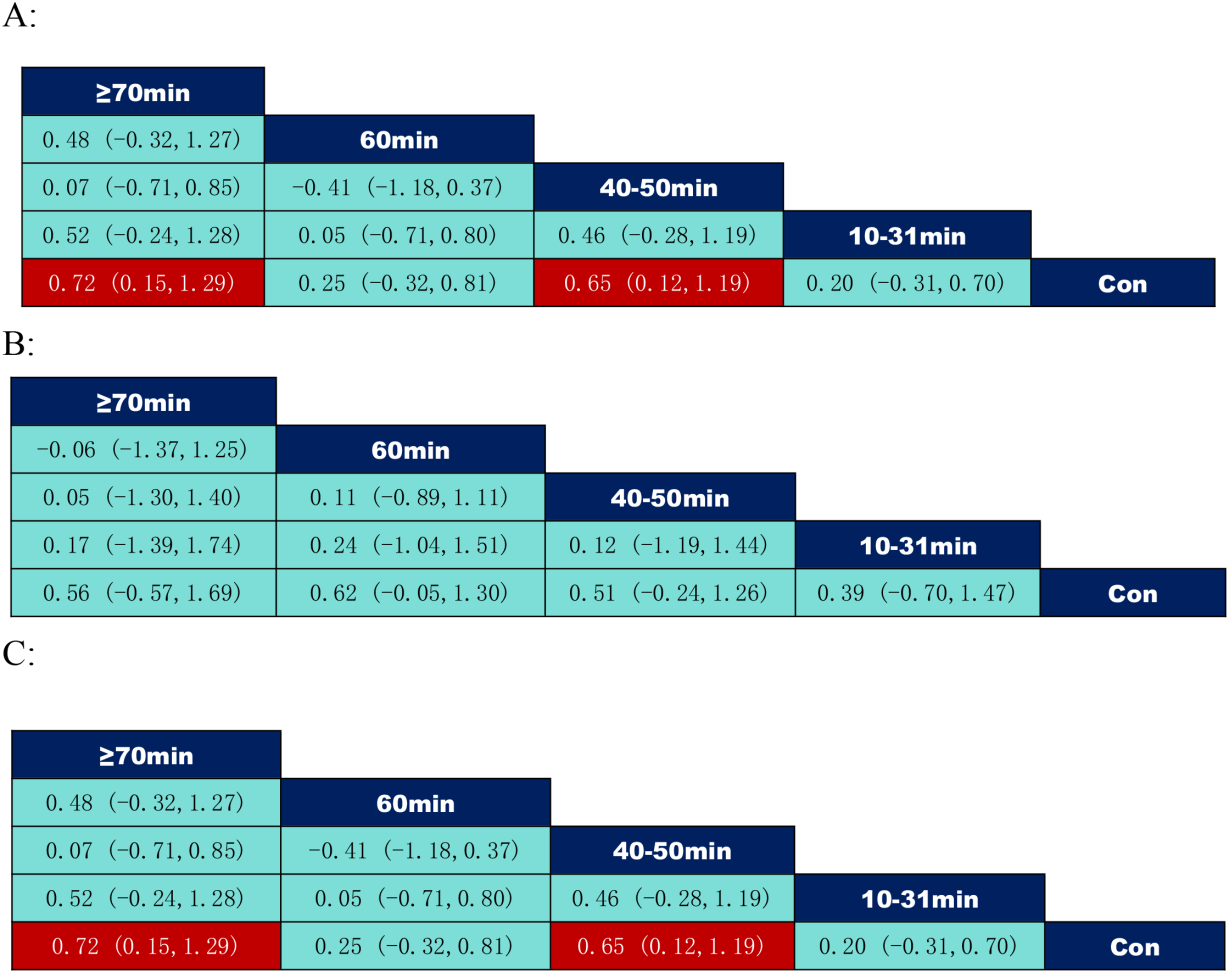
League table of pairwise comparisons of intervention effects among exercise duration elements. **(A)** Inhibitory control; **(B)** Working memory; **(C)** Cognitive flexibility.

According to the data on the duration of the exercise intervention in **Figure 7**, where inhibitory control: 40–50 minutes per session (SMD = 0.65, 95% CI: (0.12,1.19)) was significantly better than the control group; ≥70 minutes per session (SMD = 0.72, 95% CI: (0.15,1.29)) was significantly better than the control group; cognitive flexibility: 40–50 minutes per session (SMD = 0.65, 95% CI: (0.12,1.19)) was significantly better than the control group; and ≥70 minutes per session (SMD = 0.72, 95% CI: (0.15,1.29)) was significantly better than the control group.

**Figure 8:**
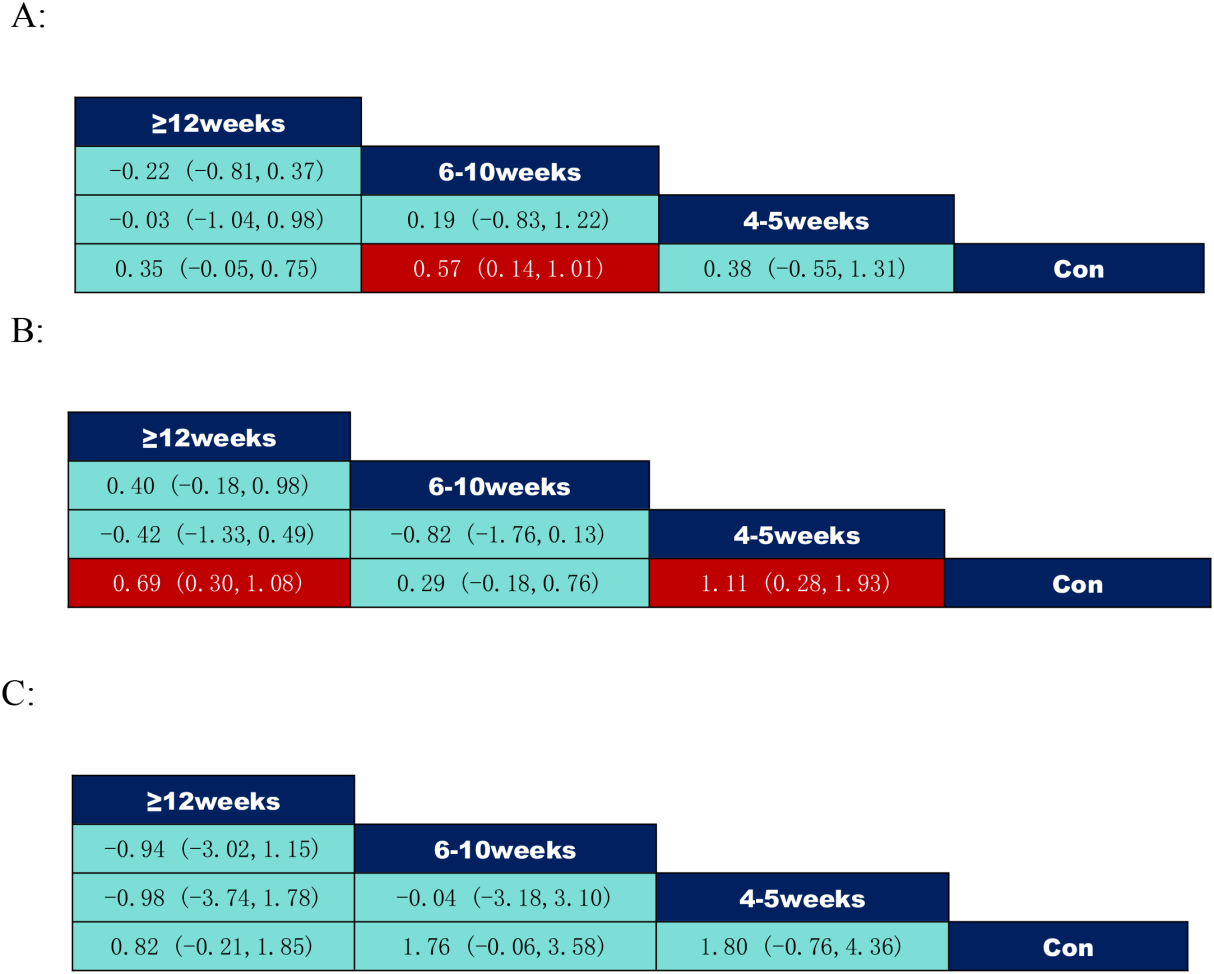
League table of pairwise comparisons of intervention effects among exercise period elements. **(A)** Inhibitory control; **(B)** Working memory; **(C)** Cognitive flexibility.

According to the data of the exercise intervention cycle in **Figure 8**, where inhibitory control: 6–10 weeks (SMD = 0.57, 95% CI: (0.14,1.01)) was significantly better than the control group; working memory: 4–5 weeks (SMD = 1.11, 95% CI: (0.28,1.93)) was significantly better than the control group, ≥12 weeks (SMD = 0.69, 95% CI: (0.30, 1.08)) demonstrated a significant improvement over the control group.

**Figure 9:**
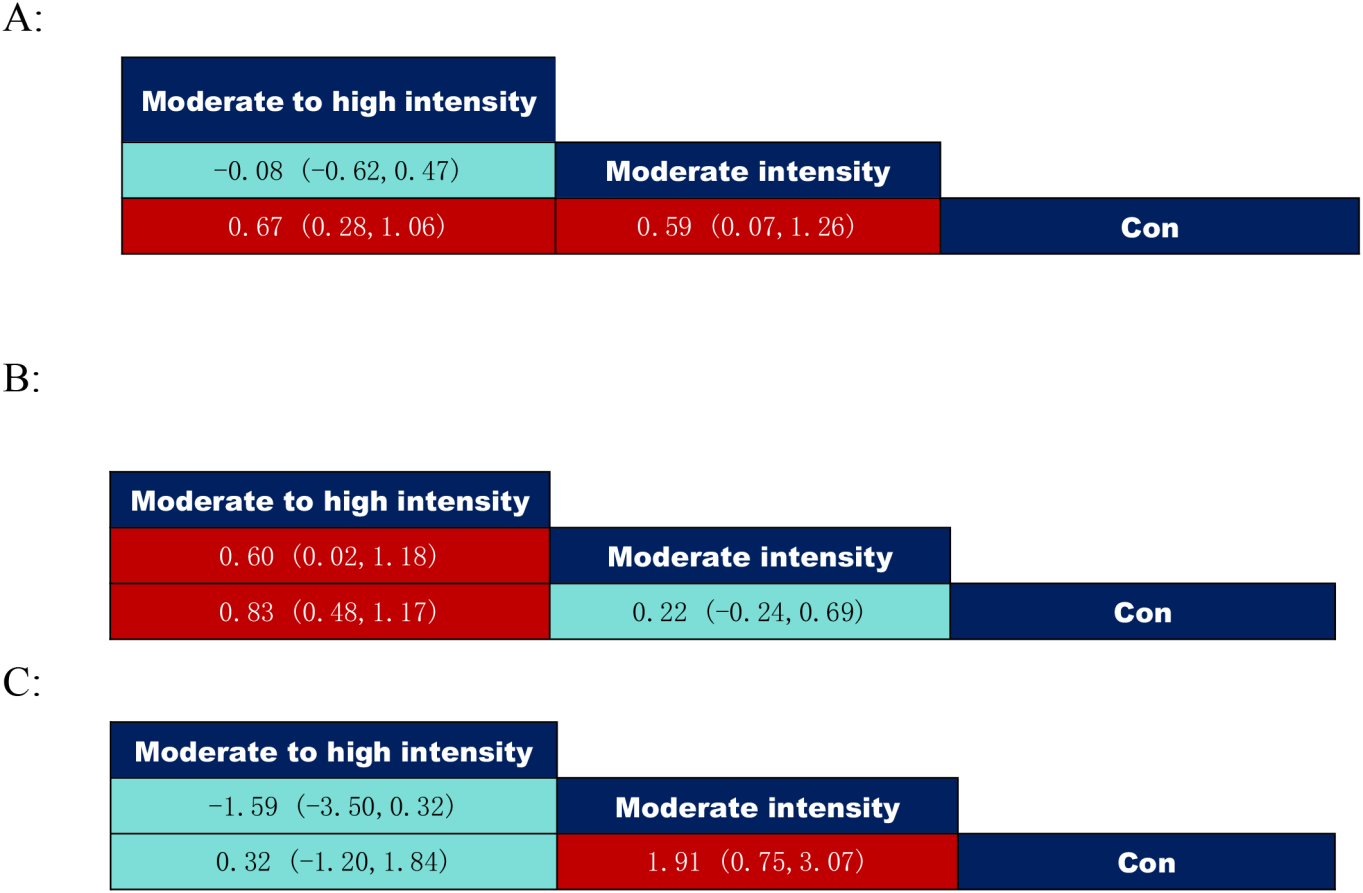
League table of pairwise comparisons of intervention effects among exercise intensity elements. **(A)** = Inhibitory control; **(B)** = Working memory; **(C)** = Cognitive flexibility.

According to the data on exercise intervention intensity in **Figure 9**, both inhibitory control at moderate to high intensity (SMD = 0.67, 95% CI: (0.28–1.06)) and moderate intensity (SMD = 0.59, 95% CI: (0.07–1.26)) significantly outperformed the control group; Working memory: Moderate to high intensity (SMD = 0.83, 95% CI: (0.48 to 1.17)) showed significantly superior outcomes compared to the control group and moderate intensity (SMD = 0.60, 95% CI: (0.02 to 1.18)); Cognitive flexibility: Moderate intensity (SMD = 1.91, 95% CI: (0.75 to 3.07)), significantly superior to the control group.

#### 3.4.3 Results of the probability ranking of the best intervention for inhibitory control

Based on the SUCRA values in **Table 5**, the ranking of exercise intervention methods is as follows: skill-based exercise (SUCRA = 95.8) > aerobic exercise (SUCRA = 51.3) > combined exercise (SUCRA = 42.2); the ranking of exercise intervention duration SUCRA is: ≥70min (SUCRA = 84.6) > 40-50min (SUCRA = 78.7) > 60min (SUCRA = 39.1) > 10-31min (SUCRA = 36.8); the SUCRA ranking of exercise intervention frequency was: 2 times (SUCRA = 95.8) > 3–5 times (SUCRA = 50.5) > 1 time (SUCRA = 45.1); and the SUCRA ranking of exercise intervention period was: 6–10 weeks (SUCRA = 80.1) > 4–5 weeks (SUCRA = 55.5) > ≥12 weeks (SUCRA = 55.3); and the SUCRA ranking of the intensity of the exercise intervention was: moderate intensity (SUCRA = 97.5) > moderate to high intensity (SUCRA = 33.6).

**Table 5:** SUCRA values for the efficacy of interventions on each element of inhibitory control.

Rank	Type	SUCRA	Duration	SUCRA	Frequency	SUCRA	Period	SUCRA	Intesity	SUCRA
1	Skill-based Exercise	95.8	≥70min	84.6	2times	95.8	6-10weeks	80.1	Moderate intensity	97.5
2	Aerobic Exercise	51.3	40-50min	78.7	3-5times	50.5	4-5weeks	55.5	Medium to high intensity	33.6
3	Combined Exercise	42.2	60min	39.1	1time	45.1	≥12weeks	55.3		
			10-31min	36.8						

#### 3.4.4 Results of probability ranking of the best intervention for working memory

Based on the SUCRA values in **Table 6**, the ranking of exercise intervention methods is as follows: aerobic exercise (SUCRA = 87.1) > skill-based exercise (SUCRA = 78.8) > combined exercise (SUCRA = 10.4); the ranking of SUCRA for the duration of exercise interventions is: 60min (SUCRA = 69.0) > ≥70min (SUCRA = 60.8) > 40 -50min (SUCRA = 59.0) >10-31min (SUCRA = 48.1); the SUCRA ranking of exercise intervention frequency was: 2 times (SUCRA = 87.6) >3–5 times (SUCRA = 55.6) >1 time (SUCRA = 52.7); and the SUCRA ranking of exercise intervention cycle was: 4–5 weeks (SUCRA = 91.7) > ≥12 weeks (SUCRA = 70.1) > 6–10 weeks (SUCRA = 33.6); and the SUCRA ranking of exercise intervention intensity was: moderate to high intensity (SUCRA = 98.7) > moderate intensity (SUCRA = 42.4).

**Table 6:** SUCRA values for the efficacy of interventions on each element of working memory.

Rank	Type	SUCRA	Duration	SUCRA	Frequency	SUCRA	Period	SUCRA	Intesity	SUCRA
1	Aerobic Exercise	87.1	60min	69.0	2times	87.6	4-5weeks	91.7	Medium to high intensity	98.7
2	Skill-based Exercise	78.8	≥70min	60.8	3-5times	55.6	≥12weeks	70.1	Moderate intensity	42.4
3	Combined Exercise	10.4	40-50min	59.0	1time	52.7	6-10weeks	33.6		
4			10-31min	48.1						

#### 3.4.5 Results of probability ranking of optimal interventions for cognitive flexibility

Based on the SUCRA values in **Table 7**, the ranking of exercise intervention methods is as follows: skill-based exercise (SUCRA = 95.5) > combined exercise (SUCRA = 57.1) >aerobic exercise (SUCRA = 46.2); the ranking of exercise intervention duration SUCRA is: ≥70min (SUCRA = 84.5) > 40-50min (SUCRA = 79.6) > 60min (SUCRA = 40.4) > 10-31min (SUCRA = 34.9); the SUCRA ranking of exercise intervention frequency was: 2 times (SUCRA = 95.8) > 3–5 times (SUCRA = 61.8) > 1 time (SUCRA = 25.1); and the SUCRA ranking of exercise intervention period was: 6–10 weeks (SUCRA = 76.3) > 4–5 weeks (SUCRA = 72.8) > ≥12 weeks (SUCRA = 45.1); and the SUCRA ranking of the intensity of the exercise intervention was: moderate intensity (SUCRA = 97.2) > moderate to high intensity (SUCRA = 55.4).

**Table 7:** SUCRA values for the efficacy of interventions on each element of cognitive flexibility.

Rank	Type	SUCRA	Duration	SUCRA	Frequency	SUCRA	Period	SUCRA	Intesity	SUCRA
1	Skill-based Exercise	95.5	≥70min	84.5	2times	95.8	6-10weeks	76.3	Moderate intensity	97.2
2	Combined Exercise	57.1	40-50min	79.6	3-5times	61.8	4-5weeks	72.8	Medium to high intensity	55.4
3	Aerobic Exercise	46.2	60min	40.4	1time	25.1	≥12weeks	45.1		
4			10-31min	34.9						

### 3.5 Sensitivity analysis

To assess the robustness of the pooled effect sizes, this study employed a leave-one-out sensitivity analysis. Upon sequentially excluding each individual study, the pooled effect sizes for inhibition control and working memory exhibited minimal fluctuation, ranging from 0.360 to 0.498 and 0.337 to 0.558 respectively. The results indicate that these two meta-analysis outcomes are relatively stable. Conversely, the effect size for cognitive flexibility ranged from 0.469 to 0.829, suggesting significant influence from individual studies and comparatively lower stability. This may relate to factors such as participant age, intervention time window, and baseline cognitive levels. Consequently, the ranking of cognitive flexibility derived from the current meta-analysis holds only exploratory significance and remains insufficient as definitive evidence for clinical decision-making.

### 3.6 Publication bias

**Figure 10:**
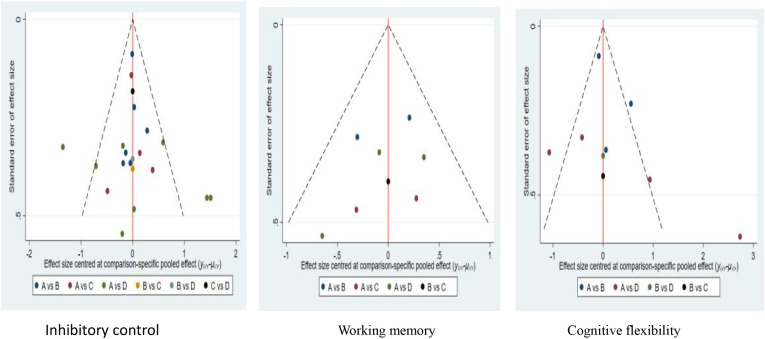
Funnel plot of intervention effects for different elements of exercise type.

Based on the visual analysis of the correction funnel plot in **Figure 10**, the effect sizes of each study exhibit a symmetrical distribution around the median of the SMD pooled values, with only a small number of points falling outside this range. The overall results indicate a low likelihood of publication bias in this study. The present study employed Egger’s method to test for publication bias in inhibitory control, working memory, and cognitive flexibility, yielding P-values of 0.082, 0.296, and 0.076, respectively. However, the Egger tests for inhibitory control (p=0.082) and cognitive flexibility (p=0.076) both approached 0.05, with only 21 studies included. Therefore, the current finding of ‘no significant publication bias’ represents only a lack of statistical significance rather than sufficient evidence, and the conclusion should be interpreted conservatively.

## 4 Discussion

The results of this network meta-analysis indicate that skill-based sports activities demonstrate the highest cumulative probability of improving inhibitory control and cognitive flexibility in children and adolescents with ADHD. Aerobic exercise, however, exhibits the most pronounced ranking advantage in enhancing working memory dimensions. Nevertheless, among the 21 outcomes examined, only 3 studies were of high quality, 6 were of low quality, and 1 was of very low quality. Sensitivity analyses revealed substantial fluctuations in the pooled effect size for cognitive flexibility when individual studies were excluded. Consequently, the present findings provide only directional guidance for exercise interventions and remain insufficient to support the formulation of precise exercise prescription dosages for enhancing cognitive function in children and adolescents with ADHD.

First, the findings of this study suggest that skill-based exercise may be the most effective means of improving inhibitory control and cognitive flexibility. However, this is inconsistent with existing research, as previous meta-analyses have indicated that aerobic exercise may be the most effective means of enhancing inhibitory control and cognitive flexibility (48). Skill-dominant activities require the brain to coordinate multiple muscle groups, and their complex, variable movement patterns exhibit a close covariation with brain activation patterns (49). Specifically, executing complex skill-based movements may stimulate synergistic coordination patterns across brain systems, thereby promoting functional optimization (50). This brain-level synergy may effectively enhance patients’ inhibitory control and cognitive flexibility (51). Skill-based sports (judo, yoga, coordination exercises) differ from combination sports and aerobic exercise in that their execution requires real-time integration of multiple sensory organs such as vision and hearing, while simultaneously adjusting movement strategies to meet immediate demands. This may exert specific stimulation on the brain’s prefrontal cortex (PFC), parietal lobe, and basal ganglia—areas central to regulating cognitive flexibility and inhibitory control. Aerobic exercise may demonstrate more pronounced effects in enhancing working memory, consistent with existing meta-analysis findings (52, 53). Furthermore, multiple studies suggest that exercise interventions primarily involving aerobic activity yield the greatest benefits for improving working memory while simultaneously reducing associated symptoms of inattentiveness (54). Functional near-infrared spectroscopy reveals that aerobic exercise enhances cortical activation in regions closely associated with working memory, such as the middle frontal gyrus and inferior frontal gyrus, providing a neural basis for working memory enhancement (55).

Regarding exercise intervention duration, sessions lasting at least 70 minutes per session are beneficial for enhancing inhibitory control and cognitive flexibility, while 60-minute sessions are more advantageous for working memory. This aligns with existing research findings, as meta-analyses have shown that exercise sessions of at least 70 minutes per session are most beneficial for inhibitory control and cognitive flexibility, while 60-minute sessions are more advantageous for working memory (56, 57). This effect arises because post-exercise serum brain-derived neurotrophic factor (BDNF) concentrations significantly increase, peaking 60–75 minutes after exercise. Through TrkB receptor-mediated mechanisms, this enhances synaptic strengthening in the prefrontal cortex and striatum (58), potentially improving patients’ inhibitory control and cognitive flexibility. This may occur because the prefrontal cortex serves as the core region for executive function, governing inhibitory control processes. BDNF-mediated synaptic strengthening optimizes neural networks in the prefrontal cortex, enhancing information processing efficiency and thereby boosting inhibitory control. This enables individuals to better suppress impulsive behaviors and focus on goal-directed tasks (59, 60). The striatum, involved in motor control, habit formation, and reward mechanisms, also plays a crucial role in cognitive flexibility (61). By enhancing synaptic function in the striatum, BDNF helps individuals flexibly switch strategies when facing complex tasks and improves their ability to adapt to new situations (62). Interventions lasting 60 minutes per session may yield optimal effects on working memory. This is likely because exercise training elevates oxygenated hemoglobin concentration in the dorsolateral prefrontal cortex by 62% within 10 minutes post-exercise, while simultaneously increasing hippocampal theta oscillation power by 3.2-fold, thereby enhancing working memory (63). However, exercise exceeding one hour may lead to excessive glutamate concentration, triggering excitotoxicity that impairs working memory maintenance (64).

Regarding exercise intervention frequency, exercising twice a week is optimal for enhancing inhibitory control, cognitive flexibility, and working memory. This twice-weekly regimen aligns with meta-analyses of existing studies (65). With sessions spaced approximately 72 hours apart, this interval allows neurons to accelerate self-repair, preparing the brain for optimal performance in subsequent workouts while potentially yielding the greatest gains in inhibitory control and cognitive flexibility (66). This is due to the dynamic equilibrium of neurotransmitter systems: the interval allows dopamine D1 receptor sensitivity to return to baseline, ensuring the basal ganglia maintain high responsiveness to new stimuli. It also aligns with synaptic remodeling cycles, where dendritic spine density in the anterior cingulate cortex continues to increase for 36–48 hours after each exercise session. The interval between interventions thus covers the active phase of synaptic structural remodeling (67). Excessive weekly intervention frequency may negatively impact children’s engagement, motivation, and exercise adherence. Therefore, a twice-weekly intervention schedule is more conducive to executive function development in children and adolescents with ADHD.

Regarding the effects of exercise intervention cycles on children’s executive functions, we found that 6- to 10-week exercise intervention cycles demonstrated more significant advantages in promoting inhibitory control and cognitive flexibility, while 4- to 5-week exercise interventions showed more pronounced effects in improving working memory. This aligns with existing research findings (60) and meta-analysis results. Exercise increases P3 wave amplitude and shortens latency, accompanied by enhanced alpha and beta band EEG oscillations. These changes collectively influence the brain’s cognitive neural mechanisms, thereby effectively enhancing children’s inhibitory control and cognitive flexibility (68). Second, the 4- to 5-week exercise intervention yielded the most pronounced improvement in children’s working memory. A potential underlying reason may be that exercise-induced enhancement of working memory exhibits a “ceiling effect.” Once exercise intervention reaches a certain threshold in boosting these cognitive abilities, further increases in duration or intensity yield diminishing returns, eventually plateauing (69). This phenomenon suggests that when designing exercise intervention programs, we must comprehensively consider the matching relationship between the exercise cycle and the target cognitive abilities to achieve optimal intervention outcomes.

Regarding intervention intensity, moderate-intensity exercise interventions may be more suitable for improving inhibitory control and cognitive flexibility, while moderate-to-high-intensity exercise interventions may be more beneficial for enhancing working memory. However, this is inconsistent with existing research findings. Previous meta-analyses suggest that high-intensity exercise may be most effective for improving patients’ working memory and inhibitory function, while moderate-intensity exercise may be optimal for enhancing cognitive flexibility (70). This study is grounded in the premise that moderate-intensity exercise (55%-70% HRmax) corresponds to the warm-up phase, gently elevating BDNF levels to establish a foundation for the prefrontal cortex and hippocampus. This may thereby promote improvements in inhibitory control and cognitive flexibility (71). Exercise at this intensity avoids neural adaptation blunting caused by excessive stimulation while preparing the physiology for subsequent high-intensity training, laying the groundwork for enhanced executive function. The progressive design of moderate-to-high intensity (55%-75% HRmax) in the middle phase, where intensity increases to 75%-85% HRmax, resembles the specialized training phase. At this point, dopamine surges, precisely enhancing the basal ganglia (72), which may subsequently improve working memory. Finally, during the high-intensity sprint phase (85%-90% HRmax), norepinephrine surges activate the anterior cingulate cortex into “rapid decision-making mode,” potentially further optimizing the efficiency of working memory execution (73).

## 5 Limitations

This study focused solely on exercise interventions without strictly controlling for daily diet or other activities. It also failed to provide stratified data on ADHD subtypes, medication status, gender, and age, thereby precluding assessment of how these key confounding factors modulate intervention effects. Secondly, approximately half of the literature failed to report details on allocation concealment and blinding methods, and lacked follow-up data, making it difficult to determine the duration of intervention effects. Thirdly, sensitivity analyses revealed that the estimated effect size for cognitive flexibility was significantly influenced by individual studies, further confirming the instability of the results. Additionally, this study only included English-language literature, potentially overlooking important research evidence from non-English-speaking countries due to language bias. Future research should conduct large-scale, high-quality randomised controlled trials (RCTs) to systematically investigate the interactive effects of ADHD subtypes, developmental stages, medication status, and gender, while tracking long-term efficacy. This will enable the development of evidence-based, individualised exercise prescriptions.

## 6 Implications for research

This study establishes a theoretical foundation for motor-based executive function interventions targeting children and adolescents with ADHD (Attention-Deficit/Hyperactivity Disorder) and confirms the positive effects of exercise interventions in enhancing executive abilities. Findings indicate that factors such as exercise type, duration, frequency, duration of the intervention period, and intensity significantly influence executive function in children and adolescents with ADHD. This discovery provides direction for future research, particularly offering a clear framework for optimizing intervention plans and developing personalized treatment strategies.

## 7 Implications for clinical practice

Given the scarcity of studies, sparse network, and overall low quality of evidence, the SUCRA probability merely suggests that ‘skill-based exercise may aid in inhibiting control/cognitive flexibility improvement, while aerobic exercise may assist in enhancing working memory’ – far from sufficient to establish precise dosage prescriptions; Should clinicians consider adjunctive interventions, these should be conducted within a framework of 60–90 minutes, twice weekly, over 4–10 weeks. Adjustments should be made dynamically based on individual subtype, medication, gender, and tolerance, with continuous monitoring of response. Any reduction in medication must be implemented stepwise by a specialist clinician, pending high-quality validation.

## 8 Implementation considerations

This study’s ‘twice weekly, 70 minutes per session’ programme may be flexibly adapted by age group: younger participants may split sessions into two 30–40 minute segments, while those with severe symptoms may begin with one 45-minute session weekly to lower exercise barriers. Accessibility varies across exercise types. While skill-based activities (martial arts, ball sports) demonstrate significant potential for improvement, they demand substantial venue space, qualified instructors, and higher financial investment. Aerobic exercises (jogging, cycling) are more suitable for large-scale implementation in resource-constrained areas or low-income households. It is recommended to prioritise aerobic exercise as the foundation, supplemented by short skill-based sessions where conditions permit. The family model should be supported by simplified instructional resources, the school model should strengthen professional teaching staff, and the clinic model should focus on children with severe conditions. Future research should validate specific adaptation strategies for resource-constrained environments.

## 9 Conclusion

This network meta-analysis suggests that different exercise types and dosage parameters may exert differential effects on various subtypes of executive function in children and adolescents with ADHD. However, limitations include the inclusion of only 21 studies, sparse network connectivity, low-quality evidence, and significant variation in effect sizes for cognitive flexibility (0.469–0.829). The identified ‘optimal dose’ serves only as a directional reference rather than a precise prescription. Clinicians should flexibly adjust within the range of 60–90 minutes per session, twice weekly, for 4–10 weeks. Future research requires dose-response trials to validate heterogeneity, extend follow-up periods, and prioritise adherence monitoring.

## Data Availability

The original contributions presented in the study are included in the article/supplementary material. Further inquiries can be directed to the corresponding author.
